# Family‐based preventive intervention for children of parents with severe mental illness: A randomized clinical trial

**DOI:** 10.1002/jcv2.12216

**Published:** 2024-02-09

**Authors:** Anne Dorothee Müller, Ida Christine Tholstrup Gjøde, Nikolaj Thams, Sidsel Ingversen, Mala Moszkowicz, Jens Richardt Møllegaard Jepsen, Lisbeth Juhl Mikkelsen, Signe Sofie Nielsen, Nicoline Hemager, Merete Nordentoft, Anne A. E. Thorup

**Affiliations:** ^1^ Faculty of Health and Medical Sciences University of Copenhagen Copenhagen Denmark; ^2^ Child and Adolescent Mental Health Center Research Unit Copenhagen University Hospital – Mental Health Services CPH Copenhagen Denmark; ^3^ Department of Mathematical Sciences University of Copenhagen Copenhagen Denmark; ^4^ Center for Neuropsychiatric Schizophrenia Research (CNSR) and Center for Clinical Intervention and Neuropsychiatric Schizophrenia Research (CINS) Mental Health Centre Glostrup University of Copenhagen Glostrup Denmark; ^5^ CORE – Copenhagen Research Centre for Mental Health Mental Health Services in the Capital Region of Denmark Mental Health Centre Copenhagen Copenhagen Denmark; ^6^ Department of Psychology University of Copenhagen Copenhagen Denmark

**Keywords:** affective disorders, family, parental mental illness, prevention, psychosis

## Abstract

**Background:**

Children of parents with a severe mental illness have an increased risk of developing a lifetime mental illness. We aimed to compare the effects of a preventive family‐based intervention, VIA Family, with treatment as usual (TAU) on these children's global functioning.

**Methods:**

Between 2017 and 2021, we conducted a pragmatic, rater‐blinded, two‐arm parallel‐group superiority trial in Denmark. Families with at least one child aged 6–12 years and at least one biological parent with schizophrenia spectrum disorder, bipolar disorder, or recurrent major or moderate depression were included. We randomly allocated 95 families with their 113 children to VIA Family or TAU (ratio 1:1). VIA Family was individually tailored and based on case management. The intervention included options for psychoeducation, parental support, and treatment for emerging child psychiatric symptoms. Blinded raters assessed children and their families at baseline and after 18 months. The primary outcome was the difference in change between groups at end‐of‐treatment in daily global functioning measured with the Children's Global Assessment Scale. Secondary outcomes were emotional and behavioral problems and days absent from school. We analyzed data blinded to allocation.

**Results:**

At post‐intervention, differences in mean change from baseline between VIA Family and TAU were non‐significant (CGAS: −1.20, 95% CI = −6.61; 4.21, *p* = 0.66), as were the differences on the secondary and exploratory outcomes.

**Conclusion:**

Contrary to our hypothesis, we did not find a superior effect of VIA Family compared with TAU. The short follow‐up period and large sample heterogeneity might explain the null findings. Therefore, a possible long‐term, preventive treatment effect has yet to be explored.

## Graphical abstract

Children of parents with severe mental illness have a high risk of developing a lifetime mental illness. In a randomized trial, we tested a family‐based preventive intervention against treatment as usual for this population. We found no differences between groups at the end of treatment.

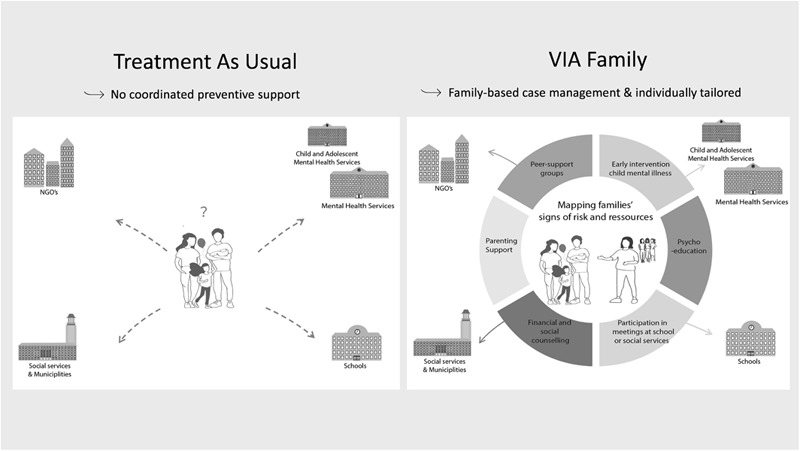

**Key points**

- Previous research has shown that children of parents with a SMI (schizophrenia, bipolar, and recurrent Major Depression) have a 55% risk of developing a lifetime mental illness compared with their peers. This evidence has called for research in preventive interventions targeting these children.
- In the VIA Family trial, we compared the efficacy of an 18‐month‐long family‐based preventive intervention with TAU.
- We did not find any significant differences in change between groups at the end of treatment.
- Despite these non‐significant findings, further research should explore the potential long‐term clinical effects of the intervention. Future studies should consider the significant heterogeneity within the group, both for intervention and trial design.

## INTRODUCTION

Preventive interventions for families with parental mental illness can potentially reduce both the prevalence and the burden of mental illness. Globally, mental illness is the leading cause of disability and suicide and comes with high social and economic costs (World Health Organization, 2022). Therefore, preventive interventions could benefit society, the economy, and the individual and the relatives. There are often early indicators of emerging or increased risk of mental illness. The strongest known risk factors for a child to develop a lifetime mental illness are being born to (genetic risk) and growing up with (environmental risk) a parent with a severe mental illness (SMI) (Rasic et al., 2014), that is, schizophrenia, bipolar disorder, and recurrent major depression (Sandstrom et al., 2019). Globally, the prevalence of parents in adult psychiatric facilities ranges from 12.2% to 45.0% (Maybery & Reupert, 2018). In Denmark, 2.2% of children (0–16 years) have at least one parent who recently (past five years) received psychiatric care for SMI (Christesen et al., 2022). Children of parents with SMI have a 30% risk of developing a SMI and a 55% risk of any other mental illness (Rasic et al., 2014). Genes account for some part of the transmission of SMI (Gronemann et al., 2023; Mullins et al., 2021; The Brainstorm Consortium et al., 2018). However, the increased risk is also caused by environmental factors (Hilker et al., 2018; McAdams et al., 2015). For example, children of parents with SMI more often live in an inadequate home environment (Thorup et al., 2022) and more often experience adverse events in childhood (Brandt et al., 2022).

Early signs of vulnerability and risk are present already during childhood. For example, children of parents with SMI more often have psychiatric symptoms (Gregersen et al., 2022; Sandstrom et al., 2020), have a lower level of psychosocial functioning (Gregersen et al., 2022; Sandstrom et al., 2019), and show impaired motor and cognitive functions (Burton et al., 2017; Hemager et al., 2018; MacKenzie et al., 2020) compared with their peers without parental SMI.

Despite this increased risk, many children with a familial predisposition do not develop a lifetime mental illness, highlighting the role of resilience. Resilience is the ability to adapt competently after exposure to adverse or stressful life events (Ungar & Theron, 2020). Protective factors build and improve a child's resilience. Internal protective factors include good social skills and self‐esteem (Hosman et al., 2004). External protective factors are in a child's environment, that is, in the family (e.g., positive parenting and a stimulating home environment), in the community (e.g., a supportive school/educational system), and at a more cultural and structural level (e.g., gender equality and economic wealth) (World Health Organization, 2022).

Family plays the most central role in these children's transmission and development of risk and protective factors (Raballo et al., 2021). Consequently, preventive interventions should target the whole family. A recent meta‐analysis showed a significant reduction of risk for the incidence of mental illness for children of parents with a mental illness who participated in preventive interventions at the end of treatment (Combined Relative Risk = 0.53, 95% Confidence Interval (CI) 0.34–0.84) (Lannes et al., 2021). Studies included in the meta‐analysis focused on several different treatment elements: Cognitive‐behavioral therapy, psychoeducation about parental mental illness, and parenting support (Lannes et al., 2021). However, because of the significant variation of needs in families, there are no one‐size‐fits‐all preventive interventions for these families (Van Santvoort et al., 2013). Consequently, the literature recommends family‐centered interventions with individual case management (Maybery et al., 2012; Wansink et al., 2015). Nevertheless, the effectiveness of these interventions is inconclusive (Maybery et al., 2012; Wansink et al., 2015). Therefore, we developed a holistic family‐based intervention, VIA Family, with a flexible approach tailored to the individual family's needs to be rigorously evaluated in a randomized design. The intervention aimed to reduce the potential impact of risk factors associated with the increased vulnerability and aimed to enhance protective factors related to resilience.

We hypothesized that a tailored and family‐based intervention compared with treatment as usual (TAU) would (a) increase global functioning (primary outcome) and (b) reduce emotional and behavioral symptoms and absence from school.

## METHODS

### Study design

The VIA Family trial is a pragmatic, rater‐blinded, randomized clinical trial in a two‐arm parallel‐group design located in the Capital Region of Denmark. The Danish Regional Committee for Health Research Ethics in the Capital Region of Denmark approved the ethical aspects of the study (Approval number H‐17000450). The Danish Data Protection Agency approved compliance with the rules on protecting personal data. We registered the study at clinicaltrials.gov (registration number NCT03497663) and published a trial protocol before data collection ended (Müller et al., 2019).

### Participants

Participants were recruited by linking data from the Danish health registers with personal data from the National Civil Registry (Pedersen, 2011) or directly from the mental health services (MHS) and social services in the Capital Region of Denmark. Eligible families had at least one child aged 6–12 years and at least one biological parent diagnosed with a SMI during the participating child's/children's life/lives (including the prenatal period). Children had to live with at least one of their biological parents within the municipalities of Frederiksberg or Copenhagen. Severe mental illness diagnoses were based on ICD‐10 or ICD‐8 criteria and defined as schizophrenia, bipolar disorder, or recurrent moderate or severe depression (see Appendix S1 for ICD‐codes). Trained mental health workers assessed all diagnoses at baseline with the lifetime diagnostic interview Schedules for Clinical Assessment in Neuropsychiatry (SCAN) (Wing, 1990). A child and adolescent psychiatrist, a trained SCAN user, confirmed all diagnoses. We excluded families recently involved in an intensive family‐based intervention addressing parental mental illness in the primary sector. Moreover, families were not eligible for the study if the primary caregiver's proficiency in the Danish language was insufficient to participate in the intervention or the data collection.

We gave all eligible parents written and oral information about the study, which included details about the potential risks of parental mental illness for their children. Before assessment commenced, all eligible parents gave written informed consent to their participation, and all caregiver(s) gave written informed their child's/children's participation.

### Randomization and masking

Families were randomly allocated (1:1) to either VIA Family or TAU, solely stratified by parental SMI diagnosis. An intervention team member randomized the participating families using the concealed electronic system: Research Electronic Data Capture (REDCap) (Harris et al., 2009). A statistician, in collaboration with the study's primary investigator, generated the sequence and stratification of randomization. Participants were enrolled and assigned to their allocation by a member of the intervention team. All outcome assessors were blinded to allocation, sequence, and block sizes. In the case of unblinding, a different assessor interviewed the participants at follow‐up. We rated clinical interviews in a team, excluding any possible unblinded assessor. It was impossible to mask participants or the intervention team due to the characteristics and duration of the treatment. Researchers were kept blinded when performing data analyses and interpreting results.

### Interventions

#### Family‐based intervention VIA Family

Families randomized to VIA Family were offered affiliation to a multidisciplinary intervention team for 18 months. We assigned all families to a case manager, who was one of four specialists in the multidisciplinary team. The team members, a social worker, a psychologist, a nurse, and a family counselor, had competencies and experience in adult MHS, child and adolescent MHS, social services, and family‐centered care. A child and adolescent psychiatrist consulted the team regularly.

**FIGURE 1:**
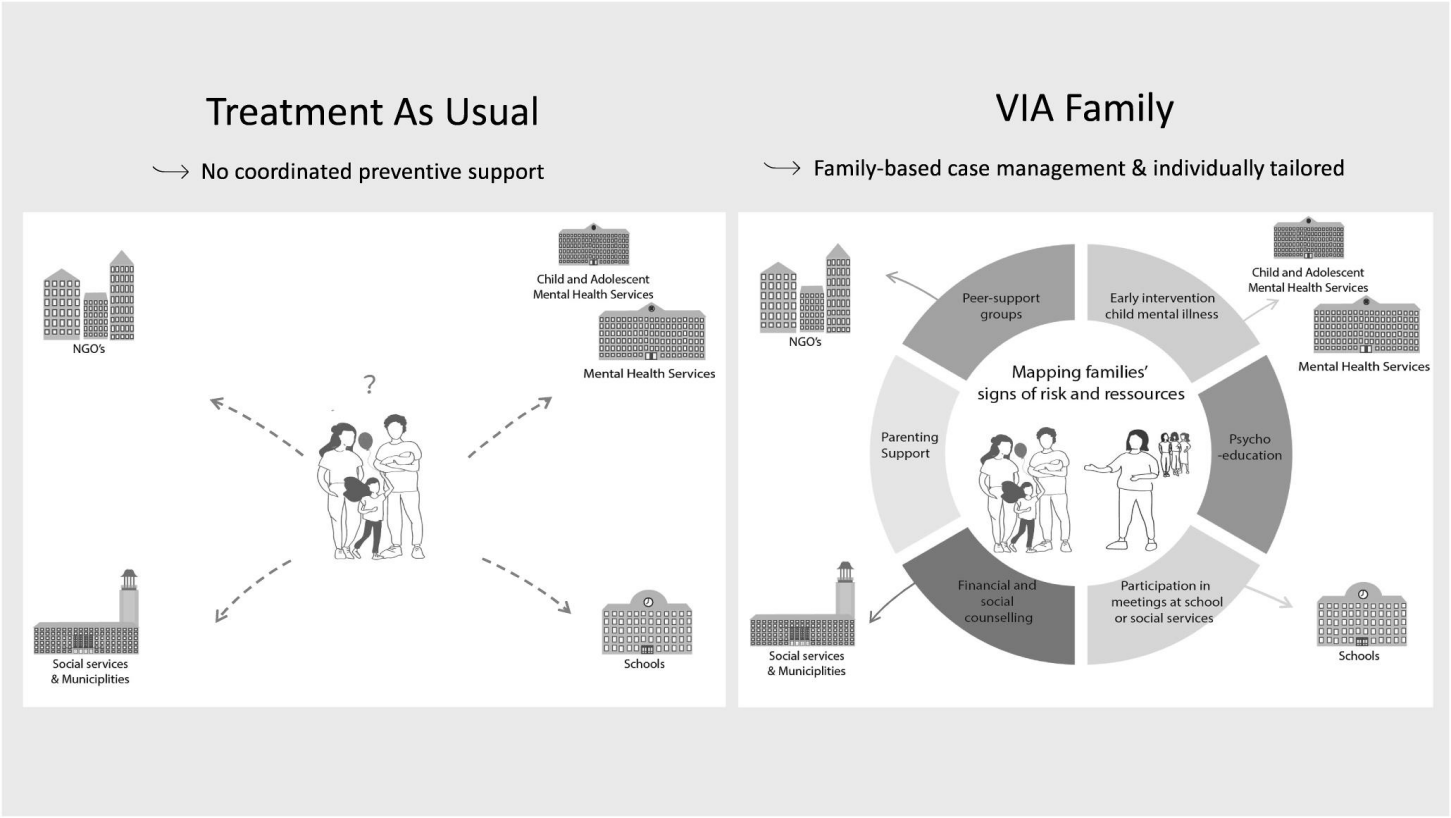
Treatment as usual (TAU) versus the tailored family‐based intervention VIA Family.

The VIA Family intervention aimed to enhance modifiable protective factors and reduce modifiable risk factors. This objective was to lower the risk of developing mental illness and associated functional impairment. Therefore, a vital characteristic of the intervention was the holistic and flexible approach focusing on each family's individual needs, motivation, and risk and protective factors. The intervention contained different elements (see Figure 1). The initial sessions included the following elements: (a) family‐centered lifeline, (b) mapping signs of vulnerability and resilience in the family (based on ‘Signs of Safety’ (Turnell et al., 2014)), and (c) family‐centered psychoeducation about mental illness or emotions. Hereafter, the family decided, in collaboration with the case manager, which elements of the intervention were relevant for them and when. The additional elements of VIA Family were: (a) help and support with parenting based on the parenting program Triple P (levels 2, 4 (standard), and 5, and Stepping Stones) (Sanders et al., 2014), (b) support groups for parents, young children, and adolescents, (c) practical support or support for pedagogical topics, school attendance, leisure time activities, or social relations, and (c) optimization of the ill parent's treatment and family's lifestyle, (d) counseling and guidance regarding financial, social, or practical support offers from the municipality, and (e) assessment of the child's and/or the parent's potential mental health problems and specialized treatment. If clinically indicated, the team referred participants directly to the MHS for further assessment or treatment. Moreover, if indicated, the team would help the family transition to support systems in the public sector (e.g., the municipality) or private sector (e.g., support groups by non‐governmental organizations (NGOs)).

Sessions were offered individually, in groups, or for the whole family. Parents could participate without their children and their children knowing about parental mental illness if they preferred to. A detailed description of the intervention elements can be found in the trial protocol (Müller et al., 2019) and the supporting Information (Appendix S2 & Table S1).

#### Treatment as usual

Families randomized to TAU were encouraged to seek relevant support in MHS, social services, and relevant NGOs.

All families, those randomized to TAU and VIA Family, received information about what kind of care for families with parental mental illness was available in the MHS in the Capital Region of Denmark, in their municipality, and the primary sector at the time of the study.

#### Treatment adherence, fidelity and training

We assessed fidelity of delivery, receipt and enactment of the VIA Family intervention following the recommendations by Bellg. et al. (Bellg et al., 2004). This included, for example, that all team members were trained equally in all the elements that required training (e.g., the parenting program Triple P) and weekly meetings for the team to discuss individual families' intervention trajectories and families' comprehension, acceptance and use of skills of the intervention. We defined adherence with intervention as a minimum of 15 contacts with the case manager and intervention team during the 18‐month intervention period. Among these, at least eight meetings had to be in person, and families had to participate in the initial sessions and at least two other elements. Team members registered each contact with the enrolled families in the REDCap system (Harris et al., 2009).

We obtained information on the use of services in usual care for both groups through parents' reports and information from the municipalities on service use related to support for the family at baseline and end‐of‐intervention.

For feasibility, the intervention elements were pilot‐tested in a small sample before the study onset (Müller et al., 2019). All four team members received regular supervision and, whenever necessary, specialized counseling by, for example, a child and adolescent psychiatrist.

### Assessments and measures

Time points for data collection were at study entry (baseline) and after 18 months (post‐intervention). All eligible children were included in the assessment. We assessed the biological sex and age of the participating children at baseline through their unique person identification number. At baseline, we did a brief screening of children's neurocognitive functioning. At both time points, trained psychologists interviewed children and their parents on demography, factors influencing psychosocial functioning, and mental health problems. See Figure 2 for an overview of all measures and time points.

FIGURE 2Outcome measures for children at baseline and after 18 months post‐intervention.

**Figure:**
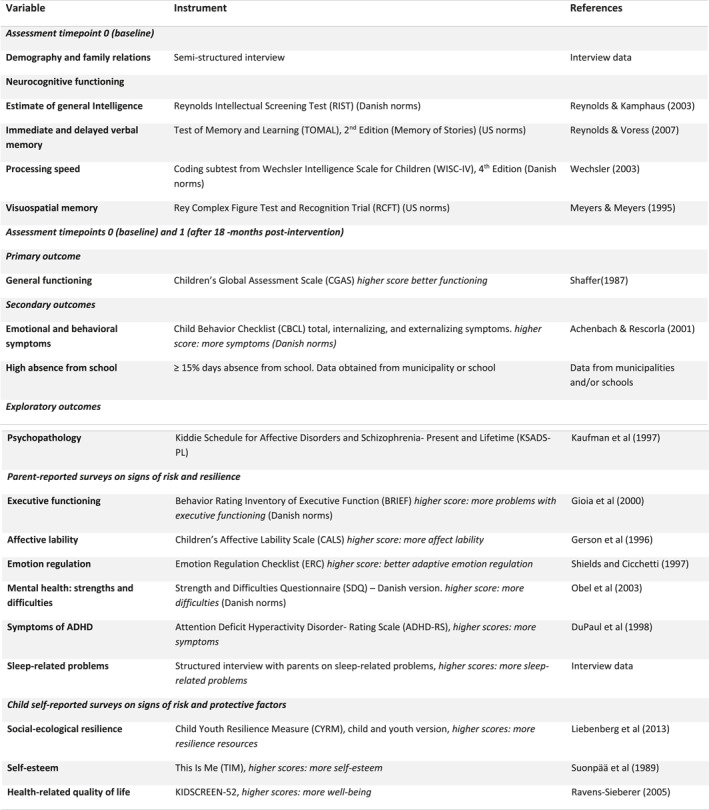

**Figure:**
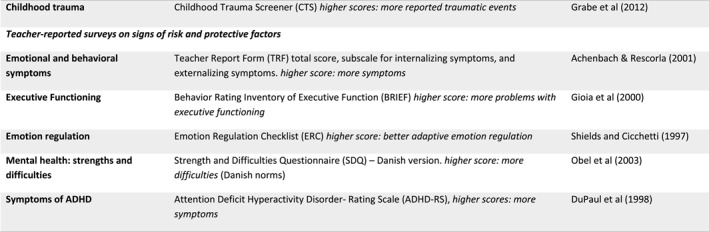

### Primary outcome

**Difference in Change in Children's Global Functioning.** We used children's global functioning as a proxy of protective and risk factors that are present within children's daily lives: at home, at school and with peers. We rated the children's global functioning with the Children's Global Assessment Scale (CGAS) (Shaffer, 1983) as part of the semi‐structured diagnostic interview Kiddie Schedule for Affective Disorders and Schizophrenia (Present and Lifetime, K‐SADS‐PL) (Kaufman et al., 1997). Children's Global Assessment Scale estimates the lowest level of functioning during the preceding month based on the child's functioning at home, at school, and with peers, based on a scale from 1 to 100 (the higher the score, the better functioning). Children's Global Assessment Scale has shown moderate inter‐rater reliability and good validity if used correctly in clinical samples (Lundh, 2012).

### Secondary outcomes

**Difference in Change in Children's Emotional and Behavioral Symptoms.** We assessed children's early signs of vulnerability, defined as emotional and behavioral symptoms, with the parent‐ and teacher‐reported questionnaires for school‐aged children from the Achenbach System of Empirically Based Assessment (Achenbach, 2001). The questionnaires measure symptoms of psychopathology at home (Child Behavior Checklist, CBCL) and at school (Teacher Report Form, TRF) based on 113 questions on a 3‐point Likert scale. A total score on dimensional psychopathology and scores on two subscales on internalizing and externalizing symptoms can be derived. It has shown good validity, and we used Danish norms (Henriksen J, Nielsen PF, Bilenberg N., 2012).

**Difference in High Absence from School.** We obtained information from the municipalities on the children's number of days absent from school 6 months before study entry and 6 months before the post‐intervention assessment (i.e., after 18 months). We collected data directly from the schools in cases of children attending private schools. We focused on ≥ 15% percentage absence from school, defined as high absence by the Ministry of Children and Education in Denmark (Børne‐og Undervisningsministeriet, 2019).

### Explorative outcomes

The child's primary caregiver, the schoolteacher, and the children themselves completed questionnaires at both time points (see Figure 2).

### Procedures

In the case of more than one caregiver, caregivers decided who was the primary to give information about the child. Trained assessors rated consentingly the primary outcome CGAS. For ethical reasons and to enhance study participation, we informed all families about the outcome of the neurocognitive and diagnostic screenings. If clinically indicated, the research team would advise families to contact their general practitioner for a referral to MHS after baseline and post‐intervention assessment. Due to ethical and legal principles, we always informed the municipalities if a family/child needed urgent help, irrespective of the randomization process.

### Adverse events

We assessed adverse events with a questionnaire for the adults and through an interview with the children and their primary caregivers. Adverse events were defined as any undesirable physical or emotional consequences participants associated with either VIA Family or TAU. In case of adverse events in the experimental group, the principal investigators decided whether to terminate the study or the participation of an individual family.

### Statistical analysis

Power calculation was based on the primary outcome CGAS. Based on a *t*‐test, we calculated that a sample size of 37 children in each group would be required to detect a significant difference between groups in a change of 10 points on the CGAS with a power of 0.90 (see trial protocol (Müller et al., 2019)). A change of 10 points is considered a large clinical effect.

All analyses were based on intention‐to‐treat (ITT) principles that included all randomized children regardless of their drop‐out status. After a qualitative assessment of reasons for drop‐out, we assumed that data were missing at random. Data were imputed using multiple imputations based on 100 imputations and 20 iterations until convergence. If data were >40% missing, we did not impute but did a complete case analysis, as suggested in the literature (Jakobsen et al., 2017). We used a linear mixed model to estimate effect sizes and test significance for the primary outcome CGAS and the secondary outcome CBCL. For school absence, we used a logistic mixed effects model. Familiarity was used as a random nested factor to account for the fact that siblings from the same family are not independent. We adjusted for age, biological sex, and baseline characteristics with significant differences (measured by a *t*‐test if continuous or chi‐square test if binary) between the groups by including them as covariates. Sensitivity analyses were performed for all primary and secondary outcomes. They included the following analyses: with and without outliers, per‐protocol, linear regression without using familiarity as random effects (i.e., assuming independent observations), and a 10% cut‐off for days absent from school.

We used the statistical software R version 4.2.2 (R Core Team, 2022) for all data analyses with the packages ‘lme4’ (Bates et al., 2015), ‘Zelig’ (Imai et al., 2008), and ‘Amelia’ (Honaker et al., 2011).

We published a plan for the statistical analyses (SAP) before data analyses as supporting information under the clinicaltrials.gov registration. The study did not include a data monitoring committee. See supporting information for changes to the protocol (Appendix S3).

## RESULTS

**FIGURE 3:**
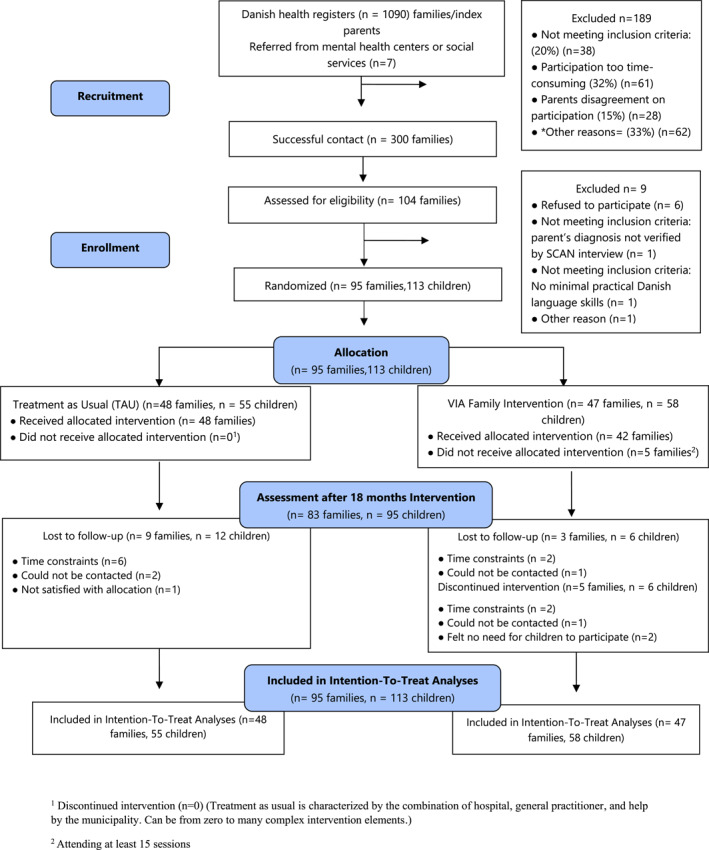
Study participation: recruitment, enrollment, randomization, and post‐intervention participation.

Between October 2017 and April 2019, we contacted 1090 eligible parents by invitation letter. Of these, 300 parents were reached by phone. The municipalities and social services referred seven families to the project. We assessed 104 families for eligibility. After screening for eligibility, nine families were ineligible; 95 families (113 children) met the inclusion criteria and were assessed. We randomized 48 families to TAU and 47 families to VIA Family. See Figure 3 for the study flow.

Children's mean age at baseline was 9.74 (SD = 1.95), 47% were girls, and the mean CGAS score was 64.67(SD = 14.22). More children in TAU had a mental disorder, lived in a single‐caregiver home, and scored lower on the verbal memory task. See Table 1 for baseline demographic and clinical characteristics of the sample.

**TABLE 1:** Baseline demographic and clinical characteristics of children, their families, and parents in VIA Family and treatment as usual (TAU).

Variable	Instrument	Treatment as usual	VIA Family	All
Children's characteristics	n	55	58	113
Biological sex, female participants^a^	n (%)	28 (50.9)	25 (43.1)	53 (46.9)
Age at baseline^a^	Mean (SD)	9.70 (2.0)	9.78 (1.9)	9.74 (2.0)
Global functioning^a^	CGAS, mean (SD)	63.78 (15.2)	65.55 (13.3)	64.67 (14.2)
Any current diagnosis^b^ ^,^ ^a^	K‐SADS‐PL, *n* (%)	21 (38.2)	15 (25.9)	36 (31.9)
Dimensional psychopathology	CBCL total problem score, T‐score (Danish norms), mean (SD)	61.51 (17.2)	56.59 (12.8)	59 (15.2)
≥15% absence from school	Data from municipalities or schools, *n* (%)	6 (10.9)	4 (6.9)	10 (8.9)
Neurocognition				
Estimate of general intelligence	RIST, scaled scores, mean (SD)	100.58 (12.3)	103.22 (13.9)	101.96 (13.2)
Processing speed	WISC‐IV *coding,* scaled scores (Danish norms), mean (SD)	8.46 (2.6)	7.56 (2.9)	8 (2.8)
Visual memory	RCFT immediate recall, T‐scores (US norms), mean (SD)	46.79 (11.3)	41.18 (12.5)	43.86 (12.3)
Verbal memory – immediate	Memory for stories, immediate recall (TOMAL‐2), scaled scores (US norms), mean (SD)	8.94 (3.0)	10.16 (2.5)	9.58 (2.7)
Verbal memory‐ delayed	Memory for stories, delayed recall (TOMAL‐2) scaled scores (US norms), mean (SD)	8.41 (3.0)	9.93 (3.2)	9.19 (3.2)
General executive functioning	BRIEF, T‐score (Danish norms), mean (SD)	52.98 (12.1)	51.73 (12.0)	52.14 (12.0)
Social responsiveness	SRS‐2, raw score, mean (SD)	118. 48 (13.2)	119.13 (17.4)	118.81 (15.4)
Family and home characteristics	n	48	47	95
Quality of home environment^c^ ^,^ ^a^	MC‐HOME/EA‐HOME, mean (SD)	46.7 (5.9)	46.4 (6.1)	46.6 (5.9)
Single parent family^d^	*parent‐reported, n* (%)	8 (16.7)	3 (6.4)	11 (11.6)
Having two parents with SMI or/and substance misuse lifetime^e^ diagnoses (solely out of families with two caregivers)	SCAN interview/patient journal, *n* (%)	8 (16.7)	5 (10.6)	13 (13.7)
Index parent's diagnosis	n	48	47	95
Lifetime^e^ SZ^a^	SCAN interview/patient journal, *n* (%)	6 (12.5)	6 (12.8)	12 (12.6)
Lifetime^e^ BP^a^	SCAN interview/patient journal, *n* (%)	12 (25.0)	13 (27.7)	25 (26.3)
Lifetime^e^ rMDD^a^	SCAN interview/patient journal, *n* (%)	30 (62.5)	28 (60.0)	58 (61.1)
Index parent with comorbid diagnosis^e^	SCAN interview/patient journal, *n* (%)	18 (37.5)	17 (36.2)	35 (36.9)
Primary caregiver's characteristics	n	48	47	95
SMI or/and substance misuse lifetime^d^ diagnoses	SCAN interview/patient journal, *n* (%)	34 (70.1)	34 (72.3)	68 (71.6)
General functioning (higher score better functioning)	PSP, mean (SD)	68.9 (16.7)	67.0 (14.4)	67.68 (15.5)
Parental stress (higher score more stress)	PSS, mean (SD)	40.1 (10.5)	39.1 (9.7)	39.64 (10.1)
Family functioning (lower score better functioning)	FAD, mean (SD)	1.9 (0.5)	1.9 (0.5)	1.89 (0.49)
Employment status parents				
Currently employed or studying^f^	*Interview, n* (%)	38 (79.2)	41 (87.2)	79 (83.2)
Educational level parents				
Primary/lower secondary	n (%)	4 (8.3)	9 (19.2)	13 (13.7)
Upper secondary, vocational, or short‐cycle tertiary	n (%)	13 (27.1)	12 (25.5)	25 (26.3)
Bachelor's degree, equivalent, or higher	n (%)	30 (62.5)	26 (55.3)	56 (58.9)
Support at baseline	n	48	47	95
Family receiving support from municipalities/MHS 6 months prior to baseline^g^	Interview/Data from municipalities, *n* (%)	12 (25.0)	11 (23.4)	23 (24.2)

*Note*: ADHD‐RS Attention Deficit Hyperactivity Disorder Rating Scale (DuPaul et al., 1998); BRIEF Behavior Rating Inventory of Executive Function (Gioia et al., 2000); CALS Children's Affective Lability Scale (Gerson et al., 1996); CBCL Child Behavior Checklist (Achenbach, 2001); CGAS Children's Global Assessment Scale (Shaffer, 1983); CTS Childhood Trauma Screener (Grabe et al., 2012); FAD Family Assessment Device (Epstein et al., 1983), HOME Home Observation for Measurement of the Environment (Caldwell & Bradley, 2003); K‐SADS‐PL Kiddie Schedule for Affective Disorders and Schizophrenia ‐ Present and Lifetime (Kaufman et al., 1997); PSP Personal and Social performance Scale (In Michalos, 2014); PSS Parental Stress Scale (Berry & Jones, 1995); RCFT Rey Complex Figure Test (Meyers & Meyers, 1995); RIST Reynolds Intellectual Screening Test (Reynolds & Kamphaus, 2003); SCAN Schedules for Clinical Assessment in Neuropsychiatry (Wing, 1990); SRS‐2 Social Responsiveness Scale‐ Version 2 (Constantino & Gruber, 2012); TOMAL‐II Test of Memory and Learning, second Edition (Reynolds & Voress, 2007); WISC‐IV Wechsler Intelligence Scale for Children, fourth Edition (Wechsler, 2003).

^a^ Data are concurrently submitted for publication in Gjøde et al. (2023).

^b^ Current Diagnosis within the last 8 weeks, excl. Elimination Disorder, Simple Phobia, Tics/Tourette's Syndrome.

^c^ For children living 50/50 with mother and father, the HOME interview was done in both homes.

^d^ Child has only one caregiver.

^e^ During child's life (including the prenatal period).

^f^ Personality disorder, substance abuse, ADHD, PTSD, or eating disorder.

^g^ Any form of employment, no minimum working hours, absence from work due to for example, illness or maternity leave included as work.

The supplementary material contains information about families' utilization of VIA Family intervention elements (Appendix, Figure S1). Moreover, it contains information on families' participation in usual care. There were no between‐group differences in families' use of services in usual care, except for more parents in the VIA Family group reporting contact to a private psychologist or psychiatrist at end‐of‐treatment (Appendix Figure S2).

### Missing data

We analyzed the data between August 2021 and October 2022. At 18 months post‐intervention, 5 out of 47 families in VIA Family discontinued the intervention for the following reasons: Intervention was too time‐consuming (*n* = 2), the family felt no need for support at the time of the study (*n* = 2), or they could not be contacted (*n* = 1). In total, 12 families were lost at the time of follow‐up assessments: 9 from TAU and 3 from VIA Family. Reasons for drop‐out were: one family was unsatisfied with randomization to TAU, eight families had time constraints, and three could not be contacted.

Missing data for primary and secondary outcomes were distributed as follows: CGAS included 17 missing values (12 in TAU and 5 in VIA Family), CBCL total score included 28 missing values (17 in TAU and 11 in VIA Family), and high absence from school included 21 missing values (10 in TAU and 12 in VIA Family). We had 58% of missing data in the teacher‐reported surveys post‐intervention.

### Primary and secondary outcomes

Results from ITT analysis on the primary outcome CGAS showed no significant differences in mean change between groups over time. The estimated mean change difference between children in VIA Family and TAU from baseline to post‐intervention was −1.2 (95% CI = −6.61–4.21, *p* = 0.66). The results remained non‐significant after adjustment for covariates. ITT analyses showed a non‐significant effect on differences in mean change between the groups in the CBCL total score (0.52, 95% CI = −4.62–5.65, *p* = 0.84), the internalizing (−0.88; 95% CI −6.85–5.1, *p* = 0.77), and the externalizing subscales of the CBCL (−0.08; 95% CI = −4.72–4.55, *p* = 0.97). Analysis of the high percentage of days absence from school showed no statistically significant difference in change between the groups (Odds Ratio (OR) = 0.30, 95% CI = 0.07–1.30, *p* = 0.11). Table 2 shows all differences in change between groups.

**TABLE 2:** Summary of treatment effect on participation in VIA Family compared with treatment as usual (TAU) derived by multiple imputations and complete case analyses from baseline to 18 months post‐intervention.

Outcome measures	Treatment as usual (*n* = 55)		VIA Family (*n* = 58)		Estimated between‐group differences/OR on mean change from baseline to postintervention (95% CI) for group VIA Family	*p*
	Baseline	Post‐intervention	Baseline	Post‐intervention		
Primary outcome (ITT)^a^						
CGAS, mean (SD)	63.78 (15.19)	69.14 (16.76)	65.55 (13.34)	69.23 (14.33)	−1.2 (−6.61; 4.21)	0.66
Secondary outcomes (ITT)^a^						
CBCL total, T‐score, mean (SD)	61.51 (17.17)	57.91 (17.06)	56.59 (12.75)	54.47 (12.01)	0.52 (−4.62; 5.65)	0.84
CBCL subscale for internalizing symptoms, T‐score, mean (SD)	62.11 (15.52)	60.53 (18.65)	56.18 (9.77)	57.32 (14.60)	−0.88 (−6.85; 5.1)	0.77
CBCL, subscale for externalizing symptoms, T‐score, mean (SD)	57.44 (17.86)	54.49 (13.49)	54.20 (13.27)	51.76 (11.82)	−0.08 (−4.72; 4.55)	0.97
≥15% days absence from school, n (%)	6 (10.90)	7 (12.73)	3 (5.17)	3 (5.17)	0.30 (0.07; 1.30)^b^	0.11
Exploratory outcomes (ITT)^a^						
Parent‐reported						
BRIEF, T‐score, mean (SD)	53.10 (12.10)	53.36 (12.77)	51.20 (11.90)	50.90 (11.83)	0.95 (−2.54; 4.45)	0.59
CALS, total raw scores, mean (SD)	10.29 (11.74)	10.51 (11.73)	7.32 (7.29)	5.87 (6.87)	−1.09 (−4.24; 1.59)	0.49
ERC, total raw scores, mean (SD)	2.81 (0.27)	2.83 (0.27)	2.87 (0.26)	2.89 (0.25)	−0.01 (−0.1; 0.08)	0.80
SDQ total difficulty score, raw score, mean (SD)	9.65 (5.90)	8.22 (6.01)	7.93 (5.89)	7.47 (5.41)	0.94 (−0.88; 2.76)	0.31
ADHD‐RS, T‐score, mean (SD)	57.69 (19.27)	54.73 (17.47)	54.48 (21.33)	53.77 (17.60)	3.69 (−2.18; 9.57)	0.22
Sleep‐related problems, n (%)	29 (52.73)	19 (34.55)	24 (43.10)	22 (37.93)	1.11 (0.26; 4.71)^b^	0.80
Child self‐report						
TIM, stanine score, mean (SD)	4.23 (1.49)	5.24 (2.19)	4.66 (1.56)	5.71 (2.13)	−0.12 (−0.84; 0.60)	0.74
KIDSCREEN‐10 (global QoL), T‐score, mean (SD)	47.19 (11.76)	52.09 (9.52)	52.15 (9.08)	53.46 (8.03)	−3.92 (−8.26; 0.43)	0.08
CTS, raw scores, mean (SD)	7.35 (3.34)	7.39 (3.50)	6.44 (2.12)	6.62 (2.31)	−0.23 (−0.86; 0.41)	0.48
Teacher‐report						
Complete‐case analyses n (%)^a^	39 (71)	21 (38)	40 (69)	25 (43)		
TRF total score, T‐score, mean (SD)	52.73 (10.79)	57.94 (18.76)	55.25 (14.66)	55.33 (12.05)	−5.43 (−15.87; 5.01)	0.30
TRF subscale for internalizing symptoms, T‐score, mean (SD)	54.52 (11.68)	57.16 (12.40)	57.88 (17.67)	53.52 (8.88)	−7.76 (−16.67; 1.16)	0.09
TRF subscale for externalizing symptoms, T‐score, mean (SD)	50.68 (13.24)	53.57 (20.75)	52.3 (16.13)	52.71 (11.60)	1.64 (−7.25; 10.54)	0.71
BRIEF‐T, T‐score, mean (SD)	51.15 (8.20)	56.38 (15.53)	53.4 (12.37)	56.7 (15.23)	−4.80 (−9.10; 8.14)	0.90
ERC‐T, total raw scores, mean (SD)	2.89 (0.29)	2.85 (0.27)	2.86 (0.3)	2.87 (0.26)	0.01 (−0.14; 0.15)	0.90
ADHD‐RS‐T, T‐score mean (SD)	48.03 (8.55)	55.63 (20.91)	50.82 (10.79)	51.59 (12.72)	−3.64 (−12.5; 5.18)	0.40
SDQ‐T total difficulty score, raw score, mean (SD)	5.85 (5.01)	7.91 (4.97)	6.73 (5.94)	6.12 (5.73)	−2.74 (−6.01; 0.54)	0.10

*Note*: ADHD‐RS: Attention Deficit Hyperactivity Disorder Rating Scale, T‐score based on Danish norms, higher scores indicate more symptoms, DuPaul et al. (1998); BRIEF: Behavior Rating Inventory of Executive Function, T‐score based on Danish norms, higher scores indicate more problems, Gioia et al. (2000); CALS: Children's Affective Lability Scale, higher scores indicate more affective lability, Gerson et al. (1996); CBCL: Child Behavior Checklist, T‐score based on Danish norms, higher scores indicate more problems, Achenbach et al. (2001); CGAS Children's Global Assessment Scale, high scores indicate better functioning (Shaffer, 1983), CTS: Childhood Trauma Screener, high scores indicate more traumatic experiences, Grabe et al. (2012), CYRM: Child and Youth Resilience Measurement, higher scores, raw scores and comparison of percentage of total mean, higher scores indicate more resilience resources, Liebenberg et al. (2013); ERC: Emotion Regulation Checklist, higher scores indicate better adaptive emotion regulation, Shields and Cicchetti (1997); SDQ: Strengths and Difficulties Questionnaire, Higher scores indicating more difficulties with mental health, Obel et al. (2003), TIM: This is Me(Suonpää et al., 1989), higher scores indicate more self‐esteem, Gerson et al. (1996); TRF: Teachers Report Form, T‐score based on Danish norms, higher scores indicate more problems, Achenbach et al. (2001).

^a^ Data are between‐group differences on mean change (SD) adjusted for age, sex, and baseline verbal memory score (immediate and recall) unless otherwise specified.

^b^ Data are OR for between‐group differences at 18 months post‐intervention adjusted for age, sex, and baseline verbal memory score (immediate and recall).

### Exploratory outcomes

ITT analyses showed no significant differences in mean change between groups in any of the explorative outcomes: executive functioning (Behavior Rating Inventory of Executive Function, BRIEF (Gioia et al., 2000): 0.95, CI 95% −2.54–4.45, *p* = 0.59), affective lability (Children's Affective Lability Scale (Gerson et al., 1996): −1.09, CI 95% −4.24–1.59, *p* = 0.49), emotion regulation (Emotion Regulation Checklist, ERC (Shields & Cicchetti, 1997): −0.01, CI 95% −0.10 to 0.08, *p* = 0.80), symptoms of ADHD (Attention Deficit Hyperactivity Disorder‐ Rating Scale, ADHD‐RS (DuPaul et al., 1998): 3.69, CI 95% 2.18–9.57, *p* = 0.22), mental health problems (Strength and Difficulties Questionnaire, total score, SDQ (Obel et al., 2003): 0.94, CI 95% −0.88–2.76, *p* = 0.31), or parent‐reported sleep‐related problems (OR = 1.11, 95% CI = 0.26–4.71, *p* = 0.80). Differences in mean changes in the child‐reported questionnaires addressing self‐esteem (This Is Me (Suonpää et al., 1989): −0.12, CI 95% −0.84 to 0.6, *p* = 0.74) or quality of life KIDSCREEN‐10 (Ravens‐Sieberer et al., 2005) total score (−3.92, CI 95% −8.26–0.43, *p* = 0.08) were non‐significant. We also did not find any significant difference in mean changes between groups in the level of child‐reported trauma (Childhood Trauma Screener (Grabe et al., 2012): −0.23, CI 95% −0.86 to 0.41, *p* = 0.48).

Complete case analyses: Due to a high number (>40%) of missing data from teacher‐reported surveys post‐intervention, we only did complete case analyses on outcomes from teacher‐reported questionnaires. Differences between the groups on the TRF (Achenbach, 2001) were non‐significant. TRF total score showed a mean difference change of −5.43 (CI 95% −15.87–5.01, *p* = 0.30), the subscale for internalizing symptoms showed a difference of −7.76 (CI 95% −16.7–1.15, *p =* 0.09), and the subscale for externalizing symptoms showed a difference of 1.65 (CI 95% −7.25–10.54; *p* = 0.70). We found no statistically significant differences in teacher‐reported children's executive functioning (BRIEF (Gioia et al., 2000)) (−4.80, CI 95% −9.10–8.14, *p* = 0.9), emotion regulation (ERC (Shields & Cicchetti, 1997)) (0.01, CI 95% −0.14–0.15, *p* = 0.9), symptoms of ADHD (ADHD‐RS (DuPaul et al., 1998)) (−3.64, CI 95% −12.5–5.18, *p* = 0.4), or mental health difficulties (SDQ (Obel et al., 2003)) (‐2.74, CI 95% −6.01–0.54, *p* = 0.10).

The sensitivity analyses did not differ from the main results of the trial. We found no significant differences between analyses with and without adjustment for age, biological sex, the child's verbal memory score, or families participating without their children (*n* = 1). Parents and children reported no adverse events associated with VIA Family intervention during and after the trial. In the TAU group, one family reported drawbacks from being informed about parental mental illness risks but receiving insufficient TAU support.

## DISCUSSION

In this trial, we compared an 18‐month preventive family‐based intervention, VIA Family, with TAU for children of parents with SMI. There were no significant differences in changes in the primary outcome, level of global functioning, or the secondary outcomes of children's emotional and behavioral symptoms and absence from school.

These findings contradict our hypotheses and previous findings in the field (Lannes et al., 2021). Evidence from preventive trials on the effect on children's global functioning is scarce. However, similar to our findings, a previous randomized controlled trial (RCT) of a preventive group cognitive behavioral intervention for youth (aged 13–18 years) with familial high‐risk for depression compared with usual care found an improvement for both groups over time, but no between‐group differences in mean change (Clarke et al., 2001).

Regarding the secondary outcome, behavioral and emotional symptoms changes, our results contradict a recent meta‐analysis on preventive interventions for children of parents with a mental illness (mood disorder, anxiety disorder, or substance use disorder) (Lannes et al., 2021). The meta‐analysis showed a small but significant effect on internalizing symptoms but no significant between‐group differences in change in externalizing symptoms. Contrary to our sample, the meta‐analysis excluded children with a mental illness diagnosis at baseline. To date, there are no results from preventive intervention RCTs for parental psychosis focusing on child outcomes (Radley et al., 2022) for comparison with our results.

### Possible explanations for null findings

Several factors could explain the null findings of this trial.

First, the end‐of‐treatment timepoint may be too early to detect the effect of a preventive intervention. A recent meta‐analysis of interventions to prevent child maltreatment showed that contrary to curative interventions, preventive interventions have a larger effect at long‐term follow‐up compared with end‐of‐treatment (Van Der Put et al., 2018). The authors explain the results with a sleeper effect of preventive interventions compared with curative interventions, that is, in preventive interventions, time passes before participants apply the newly learned skills. The long‐term effects of the VIA Family intervention are, therefore, yet to be explored. We are currently investigating the long‐term effect of the intervention for the cohort in a 3‐year post‐intervention follow‐up study.

Moreover, the intervention might have introduced changes in the participating families that are not measurable with the included instruments, for example, knowledge about risk and protective factors, reduced internalized stigma and when to seek help for early signs of vulnerability to mental illness. A qualitative meta‐synthesis of preventive interventions for this population has identified such changes after participation in preventive interventions (Radley et al., 2022). Furthermore, a meta‐synthesis determined that these particular changes were regarded as the most significant outcomes by parents with SMI (Harries et al., 2023). Especially, interventions that focused on reducing internalized stigma were associated with higher quality of life in young people (Song et al., 2023) and increased likelihood of help‐seeking (Davies et al., 2022). Moreover, a mixed‐methods study found discrepancies between qualitative findings of perceived effectiveness and quantitative results of efficacy (Maybery et al., 2019). We are currently preparing a mixed‐methods study of VIA Family participants' perception of effectiveness.

Next, TAU might be just as good as the experimental intervention, VIA Family. However, due to the complex nature of a multi‐component and individually tailored design in both interventions, it is challenging to compare the two groups (Ginsburg et al., 2021). Despite our intention to assess and ensure fidelity, the individual‐tailored approaches result in individualized intervention trajectories, which interfere with fidelity assessment and ultimately may limit conclusions about effectiveness (Ginsburg et al., 2021). Furthermore, comparing the two interventions may present additional challenges: Families randomized to TAU might have received an enhanced form of TAU due to the information on potential risks and where to seek help for early signs of vulnerability that was provided to all families after baseline assessment, regardless of randomization. Previous studies have suggested that providing information about the potential risks of parental mental illness might be sufficient to encourage parents to talk with their children about parental mental illness and to seek relevant support (Maybery et al., 2019). Psychoeducation about parental mental illness has been associated with increased likelihood of help‐seeking (Davies et al., 2022). This could be especially applicable to parents with socioeconomic resources like in our sample.

Lastly, we could not analyze differences in the children's school setting. Due to the lockdown of schools in Denmark because of the COVID‐19 pandemic during the study period, many teachers could not report on children's functioning and well‐being at school. However, a complete case analysis of teacher ratings indicated a clinically meaningful, although insignificant, difference in change in internalizing symptoms for children in the VIA Family group compared with TAU. Nevertheless, the conclusion of the results is limited due to missing data and lack of power (only 58% of teacher responses).

### Strengths and limitations

The main strength of this study is the randomized controlled design with blinded assessments, concealed allocation sequence, a non‐treatment control group, and intention‐to‐treat to account for missing data. Moreover, this is the first RCT to study an 18‐month‐long, family‐based preventive intervention of children born to parents with SMI. Another strength is the use of multi‐informants from different everyday locations of the child: parent, teacher, child, and objective participation in the experimental intervention measures.

Our study has several limitations. The small sample size does not allow for subgroup analyses. We did not include children aged younger than 6 years, which could have enabled early intervention. Moreover, for some families, parent's symptoms of SMI were in remission, which might have impacted their motivation for participation in the intervention, as described in previous studies 25.

There are some clinical implications of this trial. First, we experienced that recruitment from the MHS and the social services was difficult. The potential stigma of being a parent with a SMI prevents some families from seeking relevant support or participating in a trial (Lannes et al., 2021; Reupert et al., 2021). However, identifying the families needing support is crucial for preventive and early interventions. Mental health and social services in contact with parents with SMI should consider preventive approaches for these families. Moreover, baseline characteristics confirm the need for early identification and support for children of parents with SMI, especially for high‐risk children who show early signs of vulnerability.

Future preventive studies should consider the large heterogeneity of this population. For example, including a larger sample size will allow for sub‐group analyses. Moreover, depending on how many risk and protective factors are present around the child, a stepped‐care intervention approach could acknowledge the large heterogeneity in this population. Nevertheless, although some children have a high level of global functioning, they are still at familial high risk and should not be excluded from preventive programs. Instead, future studies should investigate screening measures for familial high‐risk children to identify who is at high risk of developing a mental illness and who is not.

In sum, despite the non‐significant findings of the current study, studies on preventive interventions for mental illness are still needed to decrease the societal, economic, and personal burden of mental illness. Future studies should include a larger sample size to account for the large heterogeneity and have a longitudinal design to measure the preventive effect on the development of mental illness and related functional impairments.

## AUTHOR CONTRIBUTIONS

**Anne Dorothee Müller**: Data curation; Formal analysis; Funding acquisition; Investigation; Methodology; Project administration; Visualization; Writing – original draft; Writing – review & editing. **Ida Christine Tholstrup Gjøde**: Data curation; Funding acquisition; Investigation; Methodology; Project administration; Visualization; Writing – review & editing. **Nikolaj Thams**: Formal analysis; Methodology; Writing – review & editing. **Sidsel Ingversen**: Data curation; Investigation; Writing – review & editing. **Mala Moszkowicz**: Data curation; Investigation; Methodology; Supervision; Writing – review & editing. **Lisbeth Juhl Mikkelsen**: Data curation; Investigation; Writing – review & editing. **Signe Sofie Nielsen**: Data curation; Investigation; Writing – review & editing. **Nicoline Hemager**: Data curation; Investigation; Methodology; Supervision; Writing – review & editing. **Merete Nordentoft**: Conceptualization; Investigation; Methodology; Supervision; Writing – review & editing. **Anne A. E. Thorup**: Conceptualization; Funding acquisition; Investigation; Methodology; Project administration; Supervision; Writing – review & editing.

## CONFLICT OF INTEREST STATEMENT

The authors have declared that they have no competing or potential conflicts of interest.

## ETHICAL CONSIDERATIONS

The Danish Regional Committee for Health 100 Research Ethics in the Capital Region of Denmark approved the ethical aspects of the study (Approval number H‐17000450).

## Supporting information

Supplementary Material

## Data Availability

The data that support the findings of this study are available on reasonable request to the corresponding author. The data are not publicly available due to privacy or ethical restrictions.
