# Supplementary material for: Family‐based preventive intervention for children of parents with severe mental illness: A randomized clinical trial
Source: JCPP Adv. 2024 Feb 9;4(3):e12216. doi: 10.1002/jcv2.12216 (PMC11472814; doi:10.1002/jcv2.12216)

# **Supplementary Material**

**Appendix S1** *Inclusion Criteria Diagnoses Codes*

We used International Classification of Diseases Version 8 and 10 (ICD-10/-8) to define inclusion diagnoses:

Schizophrenia spectrum disorder: ICD-10: F20, F21, F22, or F25, or ICD-8: 295, 297, 298.29, 298.39, 298.89, 298.99, or 301.83

Bipolar Affective Disorder: ICD-10: F30 or F31, or ICD-8: 296.19 or 296.39.,

Recurrent Major Depressive Disorder: ICD-10: F 33.1, F33.2, or F 33.3, or ICD-8: 296.0, 298.0 or 300.4

**Appendix S2** *VIA Family Intervention*

VIA Family Intervention is based on case management within a multidisciplinary team. The team members were a social worker, a pedagogue, a nurse, and a psychologist. The team had regular consultations with a child and adolescent psychiatrist. All team members had experience in mental health services, child and adolescent mental health services, and social services.

Team members were flexible and met families in their homes, at the outpatient clinic of VIA Family, or any other place where the family preferred to meet. Team members offered participation in meetings with the child’s school or the family’s municipality. Families were in contact with the case manager through phone, SMS, and online or physical meetings. In the final sessions, the team members evaluated the intervention together with the family. If indicated, the team would help the family with a transition to support systems in the public sector (e.g., the municipality) or private sector (e.g., support groups by NGOs). All elements provided were given based on the principle of least mean.

**Table S1** *Variety of Treatment Elements Offered by the VIA Family Intervention Team*

| **TREATMENT ELEMENTS** |
| --- |
| **Basic Component of the VIA Family Intervention** |
| Assignment of a case manager for the whole family |
| Introduction sessions with the case manager |
| Safety Plan |
| Psychoeducation about mental illness or emotions |
| **Additional and Optional Elements in the VIA Family Intervention** |
| (Basic) Parenting support (for children of all ages) Triple-P level 2 (seminar),  Triple-P level 3 (1-3 sessions), or parenting support |
| Triple-P level 2 (seminar), 3, 4, 5 and Stepping-stones. Individual sessions for parents (involving children in sessions, where parenting is practiced with a focus on the learned positive parenting strategies) |
| Support for collaboration between parents |
| Practical support on parenting. E.g., structuring daily activities. |
| Counseling and guidance regarding financial, social, or practical support from the municipality |
| Optimization of the ill parent’s treatment and lifestyle |
| Case manager participating in meetings with social services |
| Specialized treatment for the child’s (transient or sub-threshold) mental health difficulties. Including psychoeducation of parents on how to structure everyday life at home to support the child |
| The case manager participated in meetings with schools and/or other institutions |
| Children’s peer support groups led by case managers |
| Parent’s groups (one or both parents in a family participated) led by case managers |
| Teenager Groups |

**Appendix S3** *Changes to protocol*

*Assessment Measures Removed*. We assessed the first 30 participating children at baseline with the Multidimensional Assessment of Caring Activities (MACA) and the Positive and Negative Outcomes of Caring (PANOC). However, due to the reporting of many false positive answers, and the assessment battery being too time-consuming for the youngest participating children, the research team (ADM and IG) and primary investigator (AT) decided not to continue to assess young-carer activities with MACA and PANOC.

*Assessment Measure not Reported.* Due to a technical problem, the questionnaire on social-ecological resilience Child Youth Resilience Measure was measured differently between age groups. Therefore, we could not derive a total mean score for each treatment group. We did an exploratory analysis of differences in the mean percentage of possible the total score between groups but found no difference: -3.5; CI 95% -9.2 to 2.2; *p* = 0.23.

*Change in criteria for treatment fidelity of VIA Family.* After clinical feedback from the team working with VIA Family, we changed the criteria for adherence to protocol, to at least 8 physical meetings (before 15 contacts) which included elements from the initial sessions (family-centered lifeline, mapping of resilience and vulnerability, family-centered psychoeducation about feelings or diagnosis). Moreover, to have received VIA Family the participants had to have participated in at least two other elements of the intervention (e.g., support group and parental support). The change of fidelity criteria did not impact the sensitivity analysis on adherence to protocol.

**Figure S1**  *Families’ Use of Intervention Components in the VIA Family Allocation Group in Percentage (%)*

**
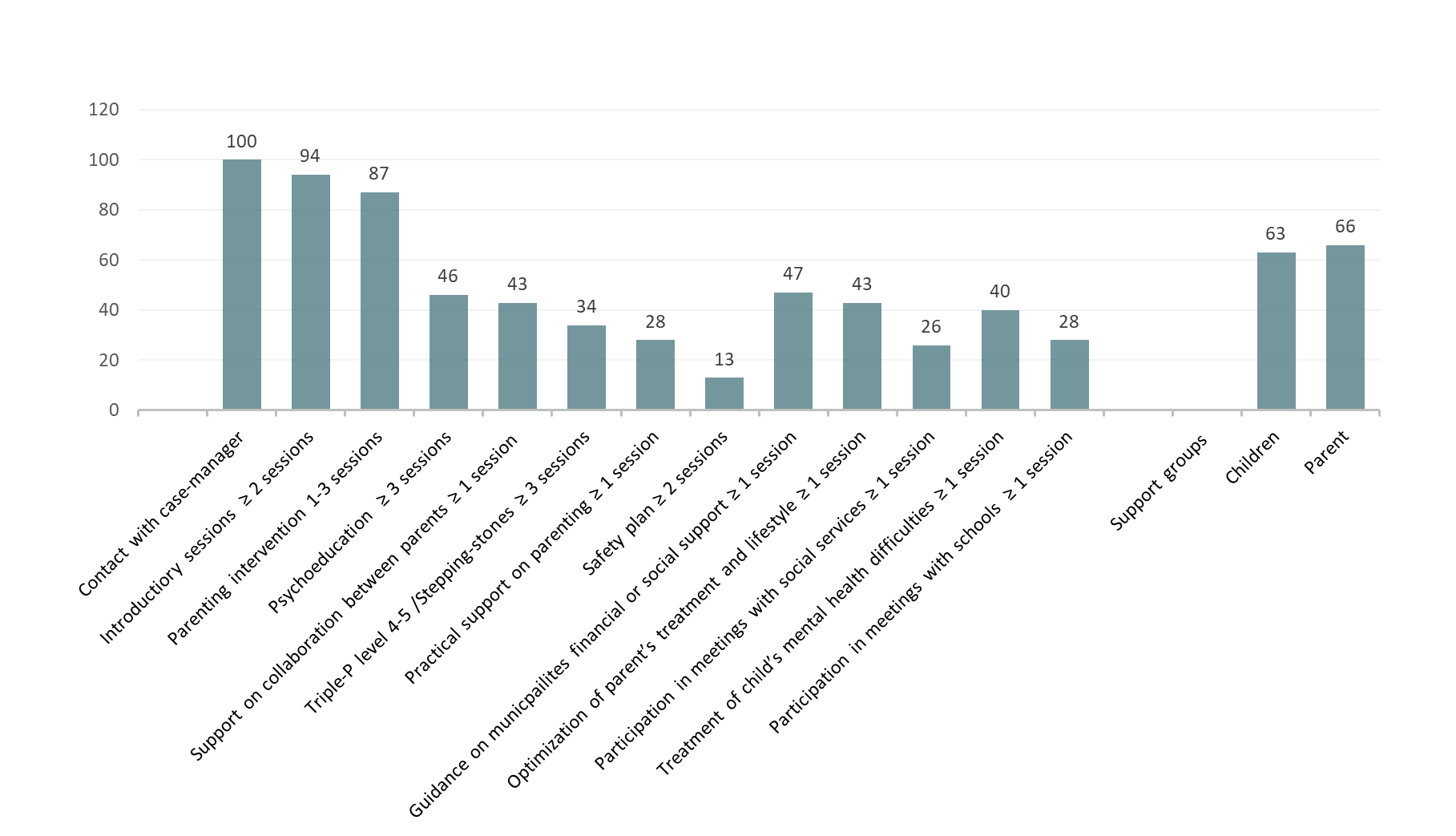
**

**Figure S2** *Families' Use of Standard Care Elements in Both Allocation Groups (VIA Family and Treatment as Usual (TAU)) in Percentage* (%)


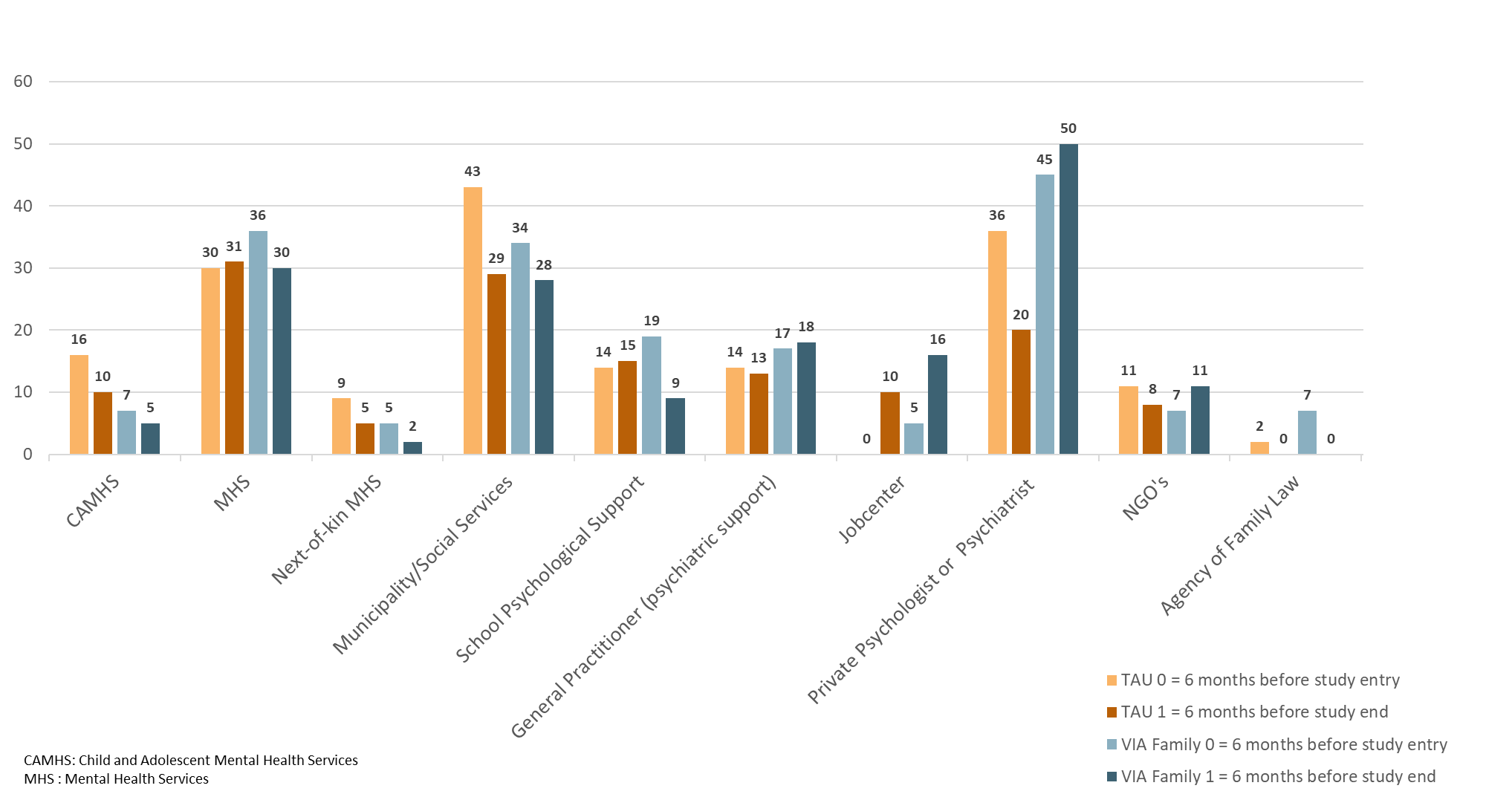


**Figure S3** *Referrals to the Social or Mental Health Services in the VIA Family Allocation Group (n=47 Families) during the 18 months intervention*


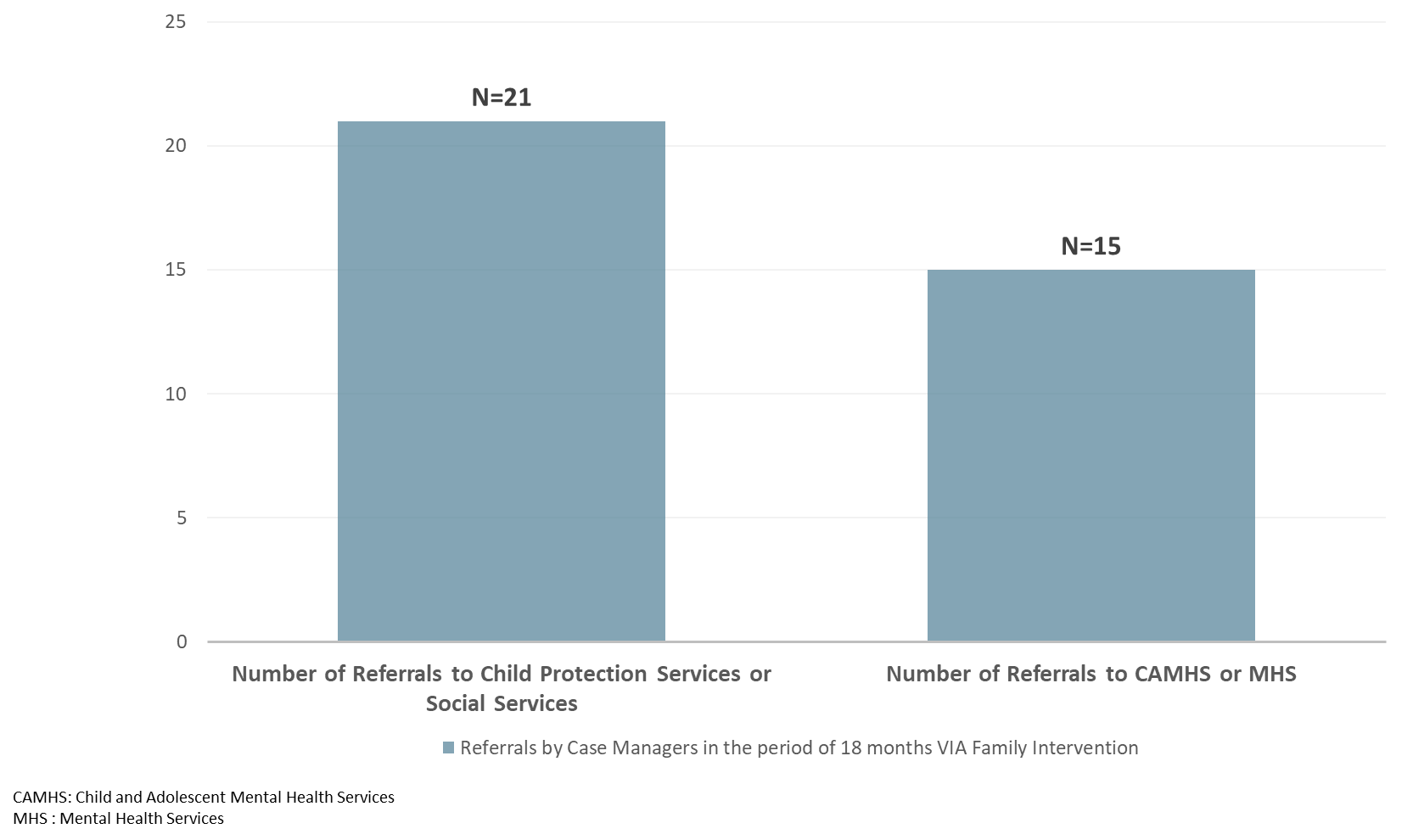

Supplement: Supplementary file 1 — Supplementary Material [file JCV2-4-e12216-s001.docx]
